# Supplementary material for: Signage Interventions for Stair Climbing at Work: More than 700,000 Reasons for Caution
Source: Int J Environ Res Public Health. 2019 Oct 8;16(19):3782. doi: 10.3390/ijerph16193782 (PMC6801962; doi:10.3390/ijerph16193782)
Supplement: Supplementary file 1 [file ijerph-16-03782-s001.pdf]

Supplementary File 1: Summary of building characteristics, baseline pedestrian behaviour, and stair climbing campaigns in the UK.

Variable	St James House	Civic Centre	Standard Court	Ferrer's Court	Town Hall	Sutton New Road	Christchurch House	Chamber of Commerce	Broadgate House
Location	Dudley	Coventry	Nottingham	Nottingham	Walsall	Birmingham	Coventry	Birmingham	Coventry
Employees	government	government	health care	commerce	government	government	government	commerce	government
Elevators	1	1	1	2	2	2	2	2	2
Floors	3	4	5	3[a]	4	4	5	6	6
Baseline Stair up	63.2%	63.1%	35.4%	52.4%	32.8%	43.5%	56.9%	39.9%	27.9%
Baseline Stair down	73.1%	80.2%	65.1%	66.7%	64.5%	61.9%	82.3%	62.9%	60.7%
Median traffic up.min^{-1}	2	2	3	3	4	3	3	2	4
(IQR, Q1:Q3)[b]	(1:4)	(1:3)	(2:5)	(2:5)	(2:5)	(2:5)	(2:5)	(1:4)	(2:6)
Median traffic down .min^{-1}	2	2	2	3	4	2	3	2	3
(IQR, Q1:Q3)	(1:4)	(1:3)	(1:3)	(2:5)	(2:6)	(1:3)	(2:4)	(1:3)	(2:4)
First campaign[c]	Calories	Calories	Calories[d]	Calories	Heart health	Fitness	Heart health	Fitness	Calories
Second campaign	Heart health	Fitness	Calories	Calories	Calories	Calories	Calories	Heart health	Heart health
Duration of phases	4 weeks	3 weeks	4 weeks	4 weeks	4 weeks	3 weeks	3 weeks	3 weeks	3 weeks

a; the distance from the ground to the first floor (7.2 m) was double the height of the other sites, and the building has been coded as three floors to reflect the increased height of the climb. b; IQR = Interquartile range, Q = quartile. c; see below for the full wording of the main messages and prompts (c.f. Thomas et al., (2015). d: For Nottingham, a message at the choice-point at the bottom of the stairs was supplemented for the second campaign with messages on subsequent floors describing the calories used to reach that floor from the ground (c.f. Lewis & Eves, 2011).

Calories main poster (A3)

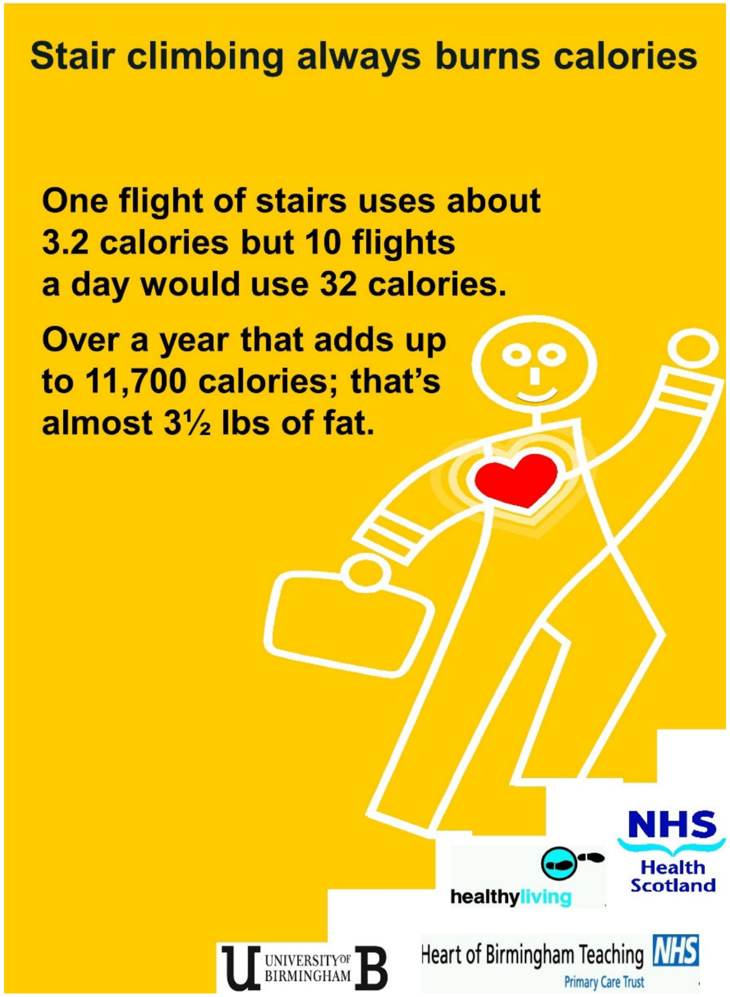

Calories prompt at lift button (A4)

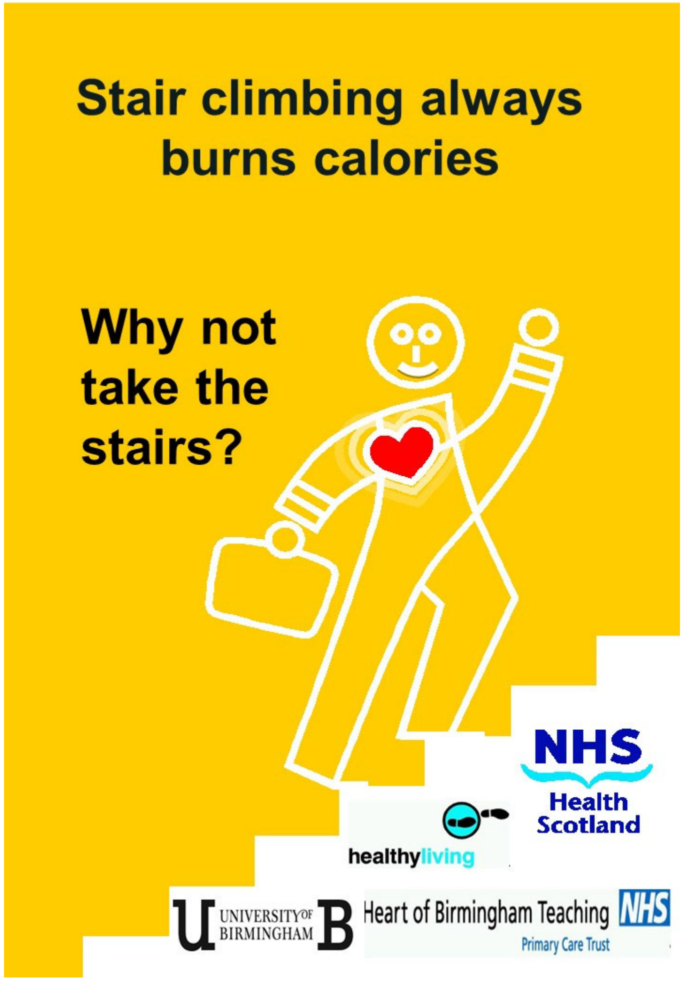

Heart health main poster (A3)

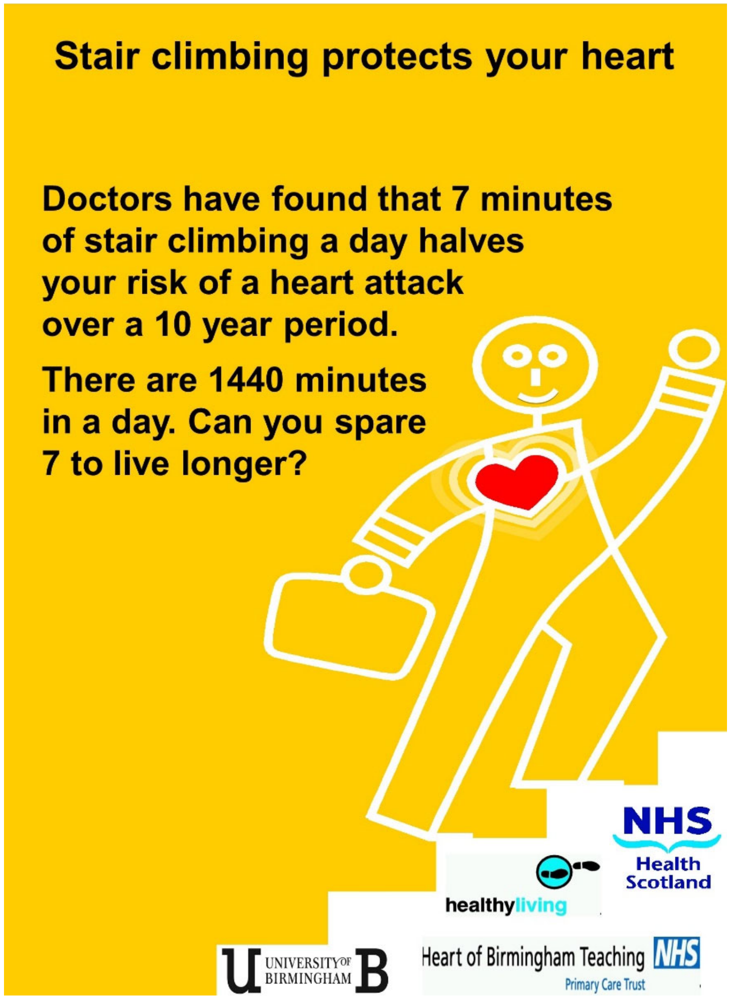

Heart health prompt at lift button (A4)

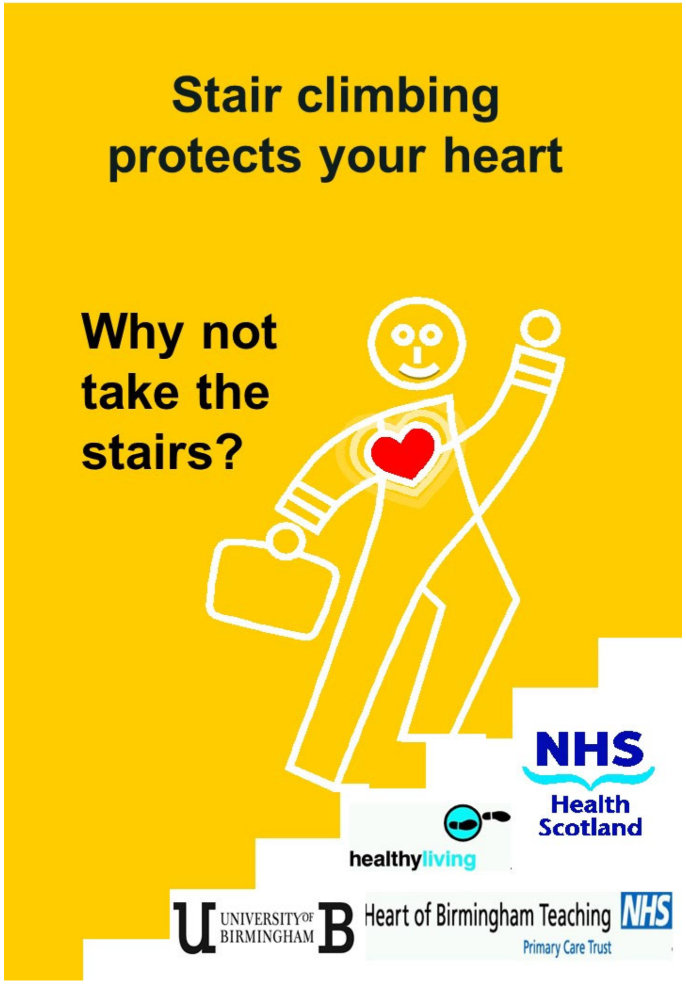

Fitness main poster (A3)

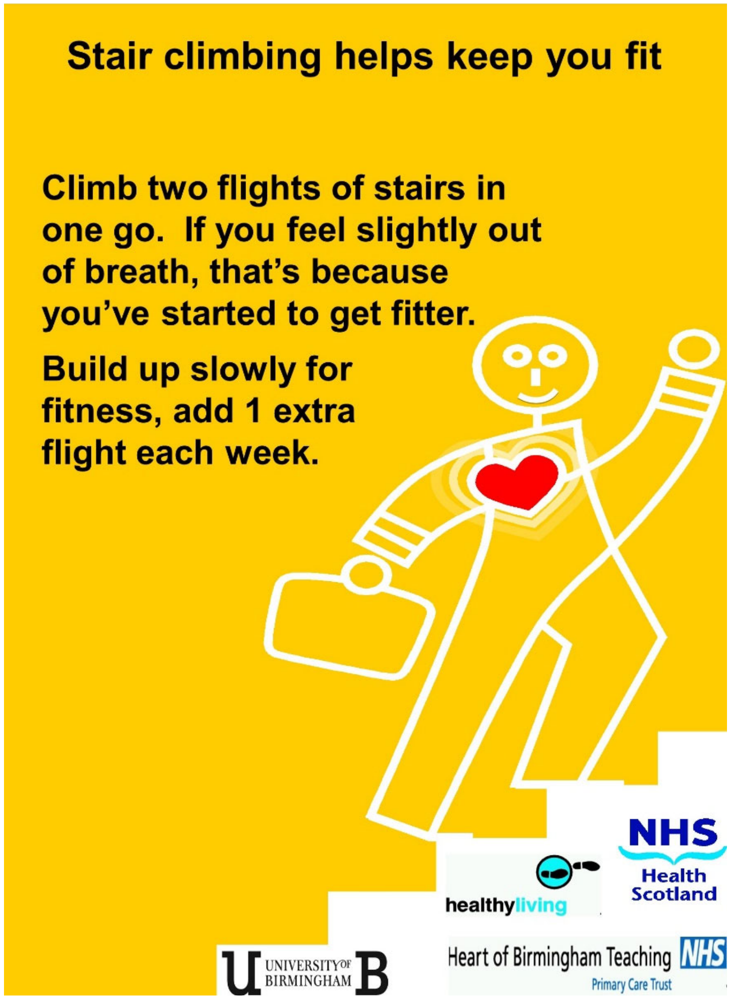

Fitness prompt at lift button (A4)

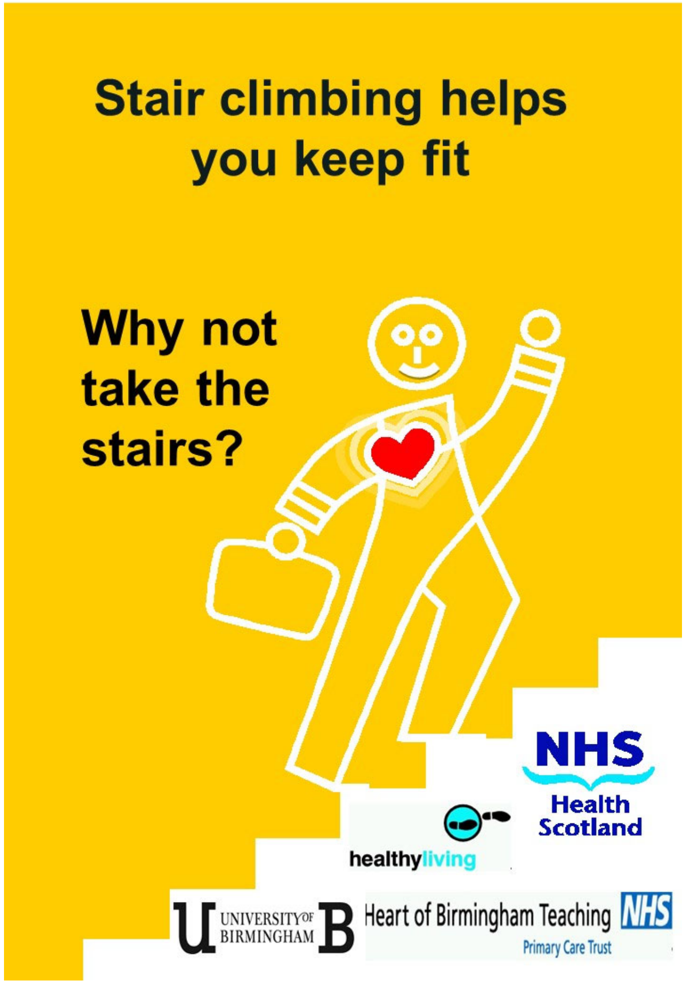

Nottingham message at choice-point (A3)

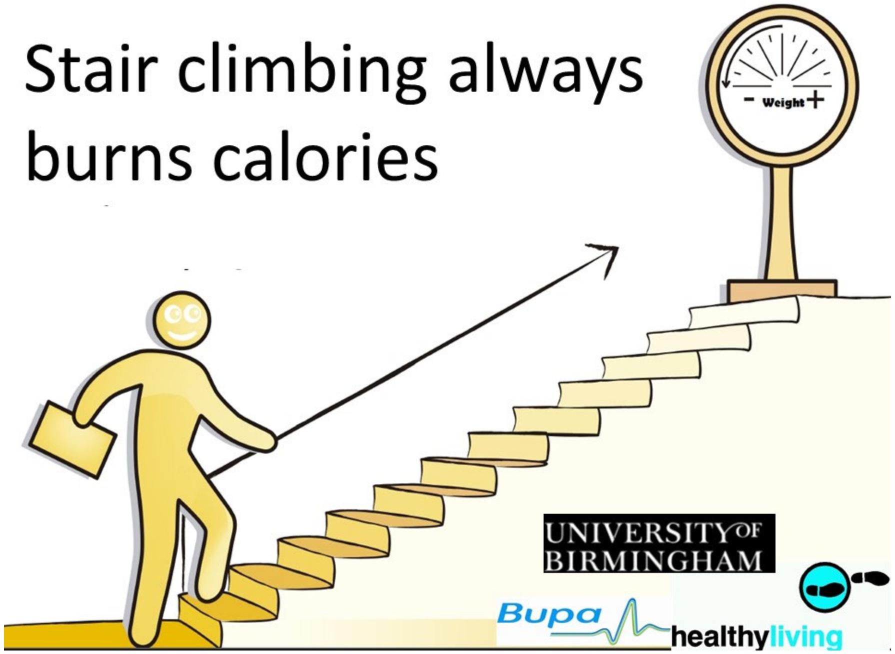

Nottingham message for third floor (A4)

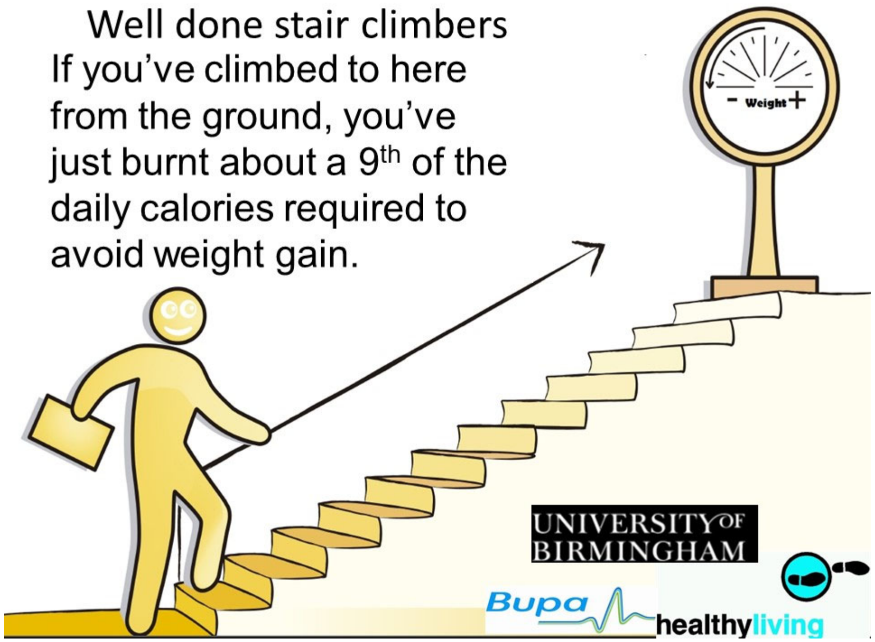

Supplementary File 2: Summary of building characteristics, baseline pedestrian behaviour, and stair climbing campaigns for Barcelona.

Variable	Almirall	Volkswagen[a] Left	Volkswagen Right	Roche[b] Entrance	Roche Canteen
Employees	commerce	commerce	commerce	commerce	commerce
Elevators	1	2	2	2	2
Floors	3	3	3	3	4
Baseline Stair up	34.5%	42.1%	37.4%	55.9%	50.9%
Baseline Stair down	53.2%	49.2%	47.4%	69.9%	67.1%
Median traffic up.30 min^{-1}	8	17	22	12	15
(IQR, Q1:Q3)	(4:14)	(9:24)	(12:35)	(6:19)	(7:29)
Median traffic down.30 min^{-1}	13	17	20	18	20
(IQR, Q1:Q3)	(8:18)	(10:23)	(12:32)	(11:25)	(12:28)
First campaign	Heart health[d]	Heart health[d]	Heart health[d]	Heart health[c]	Heart health[c]
Second campaign	Fitness[e]	Heart health[c]	Heart health[c]	Heart health[d]	Heart health[d]

a For Volkswagen, one staircase/elevator choice-point provided access to the left side of the building and one staircase/elevator choice-point access to the right. b At Roche, one staircase/elevator choice-point was at the entrance floor of the building and one at the canteen and access to the smoking area, a floor below. c Translation into Spanish of the Heart Health message used in the UK. d Catalan message for effects of stair climbing on heart health and cholesterol. e Catalan message for effects of stair climbing on fitness.

c) Translation of UK message into Spanish (A3)

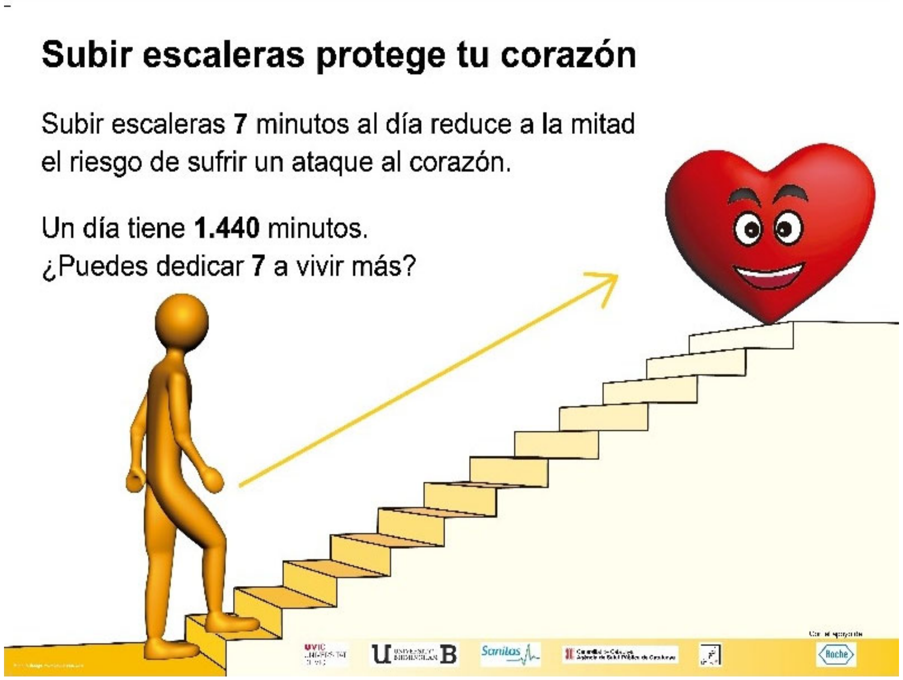

Stairwell messages (A4)
For each campaign, messages were installed in the stairwell between floors using the same poster background.

Floor 1: Climbing stairs helps lower your blood pressure
Floor 2: Keep climbing stairs. It also helps reduce cholesterol
Floor 3: Climbing stairs for 7 min during the day is very good for your heart. It also reduces abdominal fat

d) Spanish heart health message (A3)

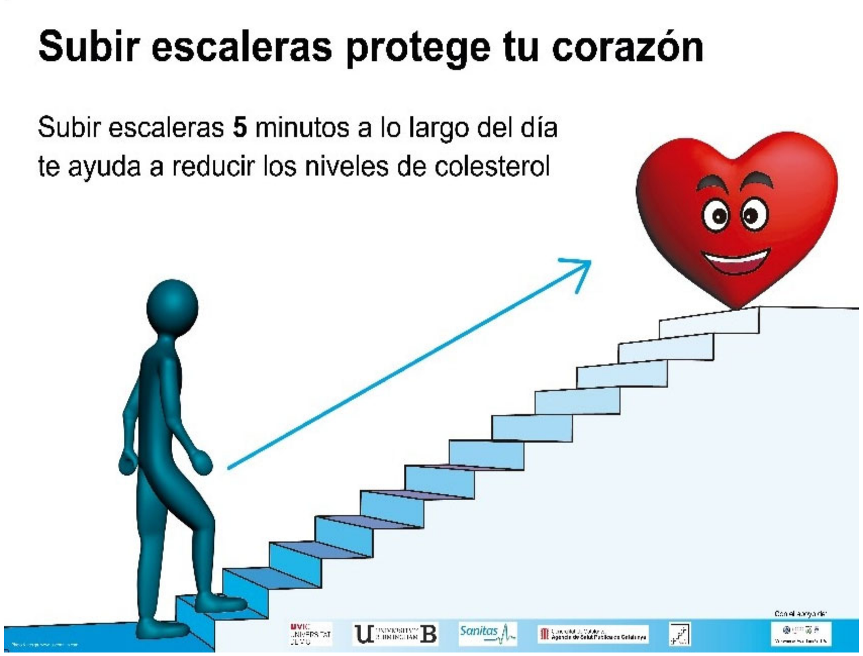

Main message translation:

Stair climbing protects your heart

5 min of stair climbing a day can reduce your cholesterol levels.

Stairwell messages (A4)
The same stairwell messages were employed.

e) Spanish fitness message (A3)

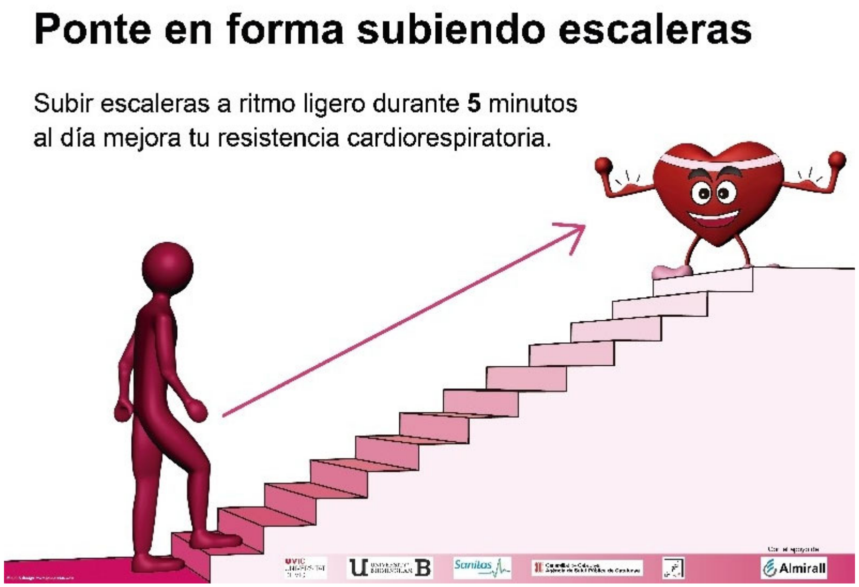

<u>Translation:</u>
Get in shape climbing stairs.

Climbing stairs at a moderate pace for five minutes a day improves your cardiorespiratory endurance.

<u>Stairwell messages on the same poster background (A4)</u>
Floor 1: Keep improving your cardiorespiratory stamina by climbing stairs.
Floor 2: Train your muscles. Climbing stairs also helps you tone your legs and buttocks.
Floor 3: Climbing stairs at work improves your fitness.

Supplementary File 3: Effects of pedestrian movement on stair use in the analyses.

To understand the effects of pedestrian movement, it is helpful to appreciate that employees within a building are making a journey from their start point to their destination. Stairs and elevators are simply hurdles to be overcome on this journey (Eves 2008, 2010). Typically, pedestrians minimize the energetic and temporal costs of their journey by selecting the shortest route (Eves et al., 2009; Gärling & Gärling, 1988; Webb & Eves, 2007). During walking, pedestrians naturally minimize energetic costs. They adopt a step width, step length, and stride frequency for walking, all of which minimize the total metabolic cost for completion of the journey (Srinivasan, 2009). Stair climbing entails at least twice the energetic cost of stair descent as body mass must be carried upstairs under the influence of gravity (Teh & Azis, 2002). Choice of an elevator minimizes the energetic costs of the journey, more so for ascent than descent. Time of the journey is also important to employees at work (Blake et al., 2008; Kerr et al., 2001a; Olander & Eves, 2011a; Thomas et al., 2015) and for pedestrians in public access settings (Eves et al., 2008; Kerr et al., 2001b); time pressure can override energy minimization (Eves et al., 2008). An elevator that is not readily available may entail increased temporal costs, and the stairs represent a quicker route to the destination (Olander & Eves, 2011a).

In the UK, ascending pedestrian traffic each minute was associated with reductions in both stair climbing and descent. This apparent consistency of effects on both directions of stair travel is misleading. For ascent from the ground floor, employees at the choice-point were exposed to the influences of other ascending employees. A waiting colleague signals that the elevator has been summoned, and joining this colleague could minimize temporal and energetic costs incurred by climbing, as well as providing an opportunity for social interaction (Eves et al., 2006). Descending employees, however, only encounter the ascending traffic at the end of their journey, (i.e., after their choice). Employees descending to the choice-point from higher floors could only be influenced by ascending traffic at locations remote from the choice-point. The effects on descent reflect aggregated behavior over a number of choice-points. If one assumes that ascending elevator users make the elevator more available on floors above the ground, effects on descending employees can be explained. Elevators signal their direction of travel and the floor at which they are located. An employee descending from a floor above may wait for an ascending elevator to arrive. Once again, temporal and energetic costs would be minimized for that individual.

In the UK, descending traffic was associated with an increase in stair use, irrespective of direction. As noted above, effects on descending employees can only reflect aggregate behavior over a number of choice-points remote from the monitored site. One simple explanation for the consistency is that descending traffic represents a proxy for 'busyness' of the building (see below). A descending employee arriving at the choice-point may see that the elevator is elsewhere in the building and choose the stairs as a quicker option.

Minute-by-minute measures of momentary traffic in the UK allowed estimates of the influences of other employees arriving at the choice-point on individual choice. For the Barcelona data, measures every 30 min provided by Solva (NL) lacked this granularity. Instead, aggregates over periods of 30 min represent overall movement within the building (i.e., 'busyness'). As a result, effects of 'busyness' in analyses for Barcelona would not be expected to mirror effects of momentary traffic tested in the UK.

Generally, 'busyness' had the opposite effect to momentary pedestrian traffic, increasing both stair climbing and descent. The more people in the building, the more likely the occupants will choose the stairs (Eves et al., 2012a, Olander & Eves, 2011a). When a building is busy, waiting times for the elevator increase, and stairs may represent the quicker route to the destination (Olander & Eves, 2011a). Increased movement throughout a building will reduce the availability of an elevator at each choice-point; stair use may be a quicker option that minimizes temporal costs despite the increased energetic cost. In Barcelona, only descending elevator traffic reduced stair climbing. Measures over 30 min will have aggregated a number of different descending elevator journeys to the choice-point; potential climbers in this time period could encounter many elevators in transit to the choice-point. The simplest explanation for a reduction in climbing is that ascending employees are willing to wait for approaching elevators that signal their approach to the choice-point. As noted earlier, stair climbing requires at least twice the energetic cost of stair descent (Teh & Azis, 2002). Less frequent stair climbing than descent was evident in both countries, consistent with the differences in energetic cost of the two directions of travel (c.f. tables 1 and 4 in the main text). These negative effects of descending elevators on stair climbing may reflect the greater reluctance to climb than descend when a mechanised alternative will be available.

References

Blake H, Lee S, Stanton T, Gorely T. Workplace intervention to promote stair-use in an NHS setting. *Int J Workplace Health Manag* 2008; 1:162-75.

Eves FF. All choices are not equal; Effects of context on point-of-choice prompts for stair climbing. *Obes Rev* 2008; **9**:83-4.

Eves FF. Point-of-decision prompts to increase stair use. *Am J Prev Med* 2010; 38:573-4.

Eves FF, Lewis AL, Griffin C. Modeling effects of stair width on rates of stair climbing in a train station. *Prev Med 2008;* 47**:**270-2.

Eves FF, Olander EK, Nicoll G, Puig-Ribera A, Griffin C. Increasing stair climbing in a train station; effects of contextual variables and visibility. *J Envir Psychol.* 2009;**29**:300-03.

Eves FF, Olander EK, Webb OJ, Griffin C, Chambers J. Likening stairs in buildings to climbing a mountain: self-reports of expected effects on stair climbing and objective measures of effectiveness. *Psychol Sport Exerc* 2012a; 13:170-6.

Eves FF, Webb OJ, Mutrie N. A workplace intervention to promote stair climbing: greater effects in the overweight. *Obes* 2006; 14:2210-16.

Gärling T, Gärling E. Distance minimization in downtown pedestrian shopping. *Environ Plan A.* 1988;20:547-554.

Kerr J, Eves F, Carroll D. Can posters prompt stair use in a worksite environment? *J Occup Health* 2001a*;* 43:205-7.

Kerr J, Eves FF, Carroll D. Getting more people on the stairs: The impact of a new message format. *J Health Psychol.* 2001b;**6**:495-500.

Olander EK, Eves FF. Elevator availability and its impact on stair use in a workplace. *J Envir Psychol* 2011a; 31:200-6.

Srinivasan M. Optimal speed for walking and running, and walking on a moving walkway. *Chaos.* 2009; **19**:026112 1-10.

Teh KC, Aziz AR. Heart rate, oxygen uptake, and energy cost of ascending and descending the stairs. *Med Sci Sports Exerc* 2002; 34:695-9.

Thomas EL, Puig Ribera A, Senye-Mir A, Greenfield S, Eves FF. Testing messages to promote stair climbing at work. *Int J Workplace Health Manag* 2015; 8:189-205.

Webb OJ, Eves FF. Effects of environmental changes in a stair climbing intervention: generalization to stair descent. *Am J Health Prom*. 2007;**22**:38-44.
